# Development and validation of a LC-MS/MS assay for pharmacokinetic studies of complement C5a receptor antagonists PMX53 and PMX205 in mice

**DOI:** 10.1038/s41598-018-26387-4

**Published:** 2018-05-25

**Authors:** Vinod Kumar, John D. Lee, Richard J. Clark, Trent M. Woodruff

**Affiliations:** 1 School of Biomedical Sciences, the University of Queensland, Brisbane, QLD 4072 Australia; 2 University of Queensland Centre for Clinical Research, the University of Queensland, Brisbane, QLD 4029 Australia

## Abstract

PMX53 and PMX205 are cyclic hexapeptide inhibitors of complement C5a receptors (C5aR1), that are widely used to study C5aR1 pathobiology in mouse models of disease. Despite their widespread use, limited information regarding their pharmacokinetics have been reported. Here, a bioanalytical method for the quantitative determination of PMX53 and PMX205 in plasma, brain and spinal cord of mice was developed using liquid chromatography-tandem mass spectrometry (LC-MS/MS) techniques. The LC-MS/MS method was validated in all three matrices according to regulatory guidelines and successfully applied to pharmacokinetic studies of PMX53 and PMX205 in C57BL/6 J mice following intravenous administration. The developed method was highly sensitive and sufficiently accurate with a lower limit of quantification within the range of 3–6 ng/ml in extracted plasma samples and 3–6 ng/g in processed tissue samples, which outperforms previously published LC-MS/MS methods. The results thus support the suitability, reliability, reproducibility and sensitivity of this validated technique. This method can therefore be applied to perform a complete pre-clinical investigation of PMX53 and PMX205 pharmacokinetics in mice.

## Introduction

An enzymatic cascade, the complement system is a vital component of the immune system creating a bridge between the innate and adaptive immune systems. This signalling pathway is omnipresent throughout the animal kingdom including invertebrates lacking a circulatory system^1^. Activation of the complement system results in terminal activation of an extremely potent complement fragment, C5a, that exhibits various immuno-regulatory and pro-inflammatory biological activities^2^. C5a binds to two known receptors, termed C5a receptor 1 (C5aR or CD88 – now referred to as C5aR1) and C5a receptor-like 2 (C5L2 or GPR77 – now referred to as C5aR2)^3^. C5aR1 is generally expressed at higher levels than C5aR2, and activation of C5aR1 enhances disease pathology, including diseases affecting the brain^2,4–7^. As such, there has been much interest in developing inhibitors to C5aR1 as therapeutic treatments for a wide range of diseases^8–10^.

The most well-studied inhibitors of C5aR1 are Ac-Phe-[Orn-Pro-dCha-Trp-Arg] (3D53 or PMX53)^11^ and hydrocinnamate-[Orn-Pro-dCha-Trp-Arg] (PMX205)^12^. These small cyclic peptidic molecules specifically target C5aR1 at nanomolar concentrations and act in a pseudo-irreversible and insurmountable manner^13,14^. PMX205 is a lipophilic analogue of PMX53 that demonstrates improved *in vivo* stability and efficacy^5,15,16^, and has been suggested as a more ideal drug candidate, particularly for neurological diseases. For example, this drug has shown beneficial effects in models of Huntington’s disease^5^, amyotrophic lateral sclerosis^4,16^, spinal cord injury^6,17^, and in reduction of memory loss in mice with Alzheimer’s disease^18,19^. Both antagonists have been used in numerous experimental inflammatory conditions for over 15 years, and oral and topical PMX53 has also been tested in early Phase I human clinical trials^20^.

Despite this extensive usage of these C5aR1 inhibitors, relatively few studies have reported the quantitative pharmacokinetic determination of these antagonists^7,13,15,21,22^. Further, none of these prior studies have reported validated LC-MS/MS methods for the quantitative determination of PMX53 and PMX205 in mice, the major species in which these compounds are used. The present research describes the development and validation of a simple, rapid, specific and sensitive LC-MS/MS method with high accuracy and precision, allowing for the quantitative determination of drug levels in plasma, brain and spinal cord of mice. This method was successfully utilised for pharmacokinetic studies of PMX53 and PMX205 in mice following the intravenous (*i*.*v*.) route of administration, and may be useful for determining the complete comparative preclinical pharmacokinetics of these drugs in mice.

## Results

### LC-MS/MS conditions and optimization

**Figure 1:**
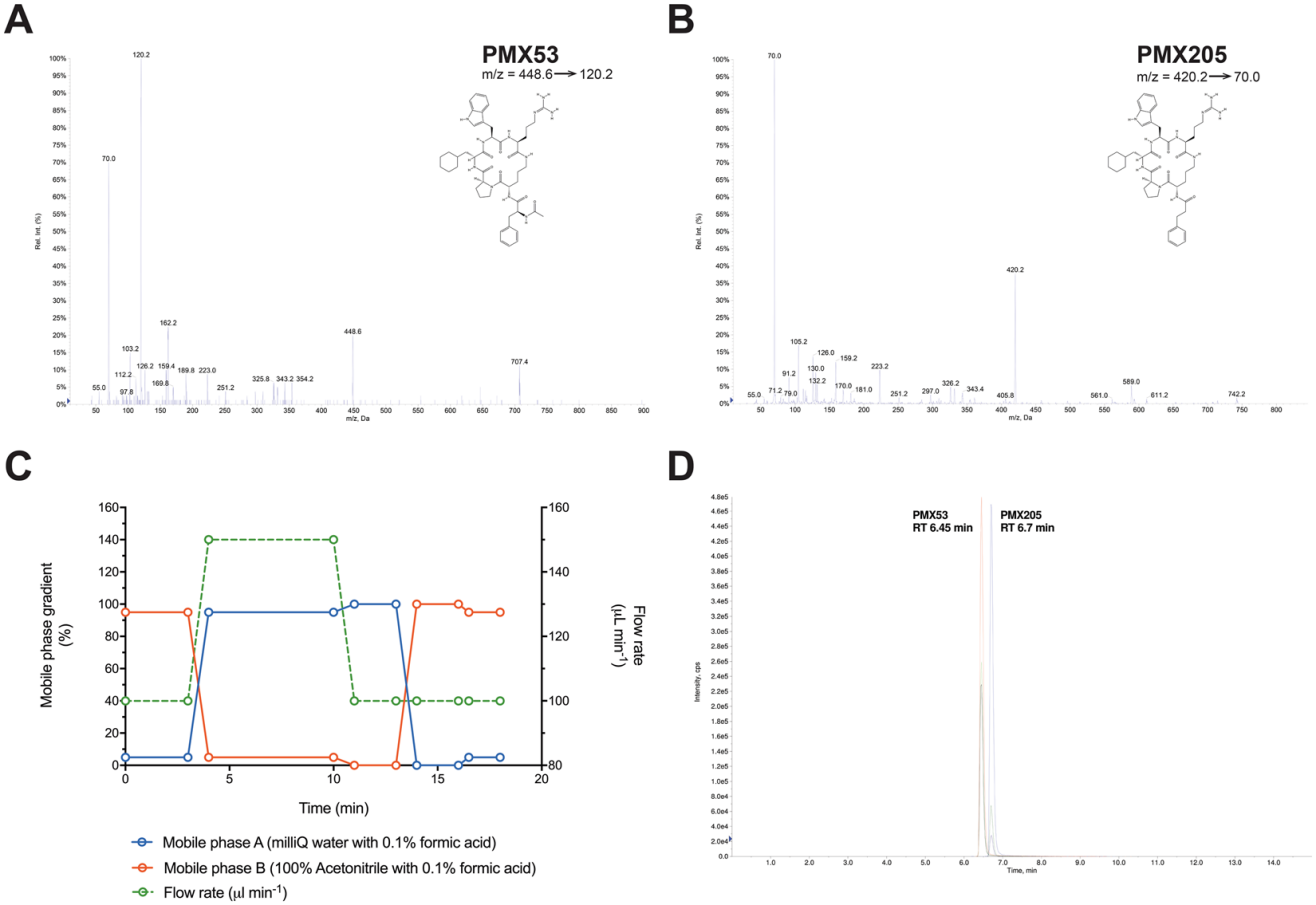
HPLC-MS/MS of PMX53 and PMX205: Product ion MS/MS scan spectra and relative intensities of fragment ions following positive (+2) electrospray ionization of PMX53 (m/z 448.6) (**A**) and PMX205 (m/z 420.2) (**B**). Developed MS method under multiple reaction monitoring mode of mass spectrometer combined with liquid chromatography gradient and flow rate conditions (**C**) for fragment ions resulted HPLC-MS/MS chromatograms of analytes as represented by overlayed chromatograms of complement C5a receptor 1 antagonists PMX53 and PMX205 (**D**).

The mass spectrometer was operated in multiple reaction monitoring (MRM) scan mode for identification of an ideal ionization state for fragmentation. Fragmentation was not achieved for both PMX53 and PMX205 in +1, −1 and −2 charge ionization mode. In the +2 ionization state, PMX53 and PMX205 underwent fragmentation resulting in formation of three main fragments. Compound-dependent and source-dependent mass spectrometer conditions were optimized using automatic optimization by infusion and flow injection analysis (FIA). The optimized compound dependent instrument parameters for PMX53 and PMX205 with their MRM transitions are summarized in Table 1. Fragmentation pattern and relative intensities of fragment ions are shown in Fig. 1A,B. For quantitative purposes, one mass/charge (m/z) transition per analyte, i.e. 448.6 → 120.2 for PMX53 and 420.2 → 70.0 for PMX205, were monitored. Additional transitions were monitored for qualification purposes, i.e. for PMX53 m/z transition of 448.6 → 70.0 and 448.6 → 162.1 and for PMX205 420.2 → 105.2 and 420.2 → 105. For both analytes, the ion spray voltage, collision cell entrance potential and source temperature/auxiliary gas temperature were set at 5000 V, 20 V and 550 °C respectively. Nebulizer gas (ion source gas 1, GS1) and auxiliary gas (ion source gas 2, GS2) were set at 70 psi. The curtain and collision-activated dissociation gases were set at 20 and 5 on an arbitrary scale.

**Table 1:** Optimized MS parameters for PMX53 and PMX205.

Analyte	ID	Q1 (Da)	Q3 (Da)	Dwell time (msec)	DP (V)	EP (V)	CEP (V)	CE (V)	CXP (V)
PMX53	Fragment 1 (Quantifier)	448.6	120.2	150	46	4	20	53	4
	Fragment 2 (Qualifier 1)	448.6	70.0	150	46	4	20	73	14
	Fragment 3 (Qualifier 2)	448.6	162.1	150	30	7	20	29	4
PMX205	Fragment 1 (Quantifier)	420.2	70.0	150	30	4	20	53	6
	Fragment 2 (Qualifier 1)	420.2	105.2	150	26	2.5	20	55	12
	Fragment 3 (Qualifier 2)	420.2	126.0	150	26	7	20	53	4

Q1, mass of analyte in (+2) ionization state; Q3, fragment mass; DP, declustring potential; EP, entrance potential; CEP, collision cell entrance potential; CE, collision energy; CXP, collision cell exit potential.

LC conditions were optimized to obtain a short run and better chromatographic separation of PMX53 and PMX205 by using various mobile phase concentrations. After several LC-MS/MS method trials, a mobile phase under binary gradient condition (Fig. 1C) of mobile phase A (milliQ water containing 0.1% formic acid) and mobile phase B (acetonitrile containing 0.1% formic acid) with variable flow rate was selected for simultaneous determination of PMX53 and PMX205 without interference from each other resulting in better resolution.

Using these optimized LC-MS/MS parameters, the retention time of PMX53 and PMX205 was approximately 6.5 min and 6.7 min respectively with a total run time of 18 min including the washing phase and equilibrium phase (Fig. 1D). Due to unavailability of radiolabelled analytes, it was difficult to find an ideal internal standard to be used in validation and analytical experiments. The chemical structure of PMX53 and PMX205 are closely related to one another and with both analytes having an adjacent retention time, this makes them ideal candidates to act as an internal standard for each other. Consequently, PMX53 was used as the internal standard for PMX205 experiments and vice versa.

### Selectivity and specificity

Chromatograms of processed blank matrices, matrices spiked with analytes and samples from mice administered with drug candidates are shown in Supplementary Figs 1 and 2. Lack of additional specific peaks of endogenous substances was observed in blank matrices indicating an absence of interference from endogenous components for the quantification of PMX53 and PMX205. The unchanged peak shape and retention time of PMX53 and PMX205 in matrices spiked with low quality control (LQC) compared to samples obtained from mice *i*.*v*. administered with drug candidates, indicates the reliability of LC-MS/MS method in terms of selectivity and specificity.

### Linearity of calibration curves and sensitivity

For the bioanalytical method validation, the calibration curve parameters for PMX53 and PMX205 in different matrices were determined on two consecutive days as summarised in Supplementary Table T1. Calibration curves were linear over the selected concentration range in different matrices. All curves with an average correlation coefficient r > 0.99 were considered for this analysis. The mean of six standard curves in plasma, brain homogenate and spinal cord homogenates were used in the validation of the bio-analytical method. Following validation guidelines, deviation of ±15% for all quality controls (QCs) and ±20% for lower limit of quantification (LLOQ) from the theoretical value was used as an acceptance criterion. Additionally, Supplementary Table T1 summarizes the lower limit of detection (LLOD) and LLOQ values for determination of PMX53 and PMX205 in various matrices. The results support that the developed method was sufficiently accurate and sensitive with LLOD and LLOQ within the range of 1.76–5.35 ng/ml for PMX53 and 1.23–3.73 ng/ml for PMX205 in extracted plasma samples. Additionally, lower limits of detections and quantifications of developed method in processed tissue samples are within the range of 2.75–6.31 ng/g for PMX53 and 1.95–5.9 ng/g for PMX205.

### Accuracy and precision

Intra-day and inter-day % relative standard deviation and % relative error for both analytes in various matrices were within acceptable limits (i.e. ±15%) (Table 2) for the validation of bioanalytical methods as per The USA Food and Drug Administration (FDA) guidelines^23^. These results favour the accuracy and precision of this analytical method over the range of the assay.

**Table 2:** LC-MS/MS method’s accuracy and precision for PMX53 and PMX205 determination.

	Matrix	Spiked conc.	Intra-day (*n* = 6)			Inter-day (*n* = 6 × 3)		
			Measured conc. (mean ± SD)	Precision (RSD, %)	Accuracy (RE, %)	Measured conc. (mean ± SD)	Precision (RSD, %)	Accuracy (RE, %)
PMX53	Plasma	LQC (6.25 ng/ml)	6.24 ± 0.17	2.75	−0.06	6.20 ± 0.28	4.56	−0.87
		MQC (25 ng/ml)	24.81 ± 0.46	1.85	−0.74	24.96 ± 1.46	5.84	−0.17
		HQC (200 ng/ml)	199.9 ± 1.23	0.62	−0.03	198.83 ± 1.9	0.96	−0.59
	Brain	LQC (6.25 ng/g)	6.30 ± 0.46	7.36	0.73	6.24 ± 0.3	2.70	0.46
		MQC (25 ng/g)	24.7 ± 0.78	3.17	−1.20	25 ± 1.62	2.06	−0.68
		HQC (200 ng/g)	199.3 ± 5.48	2.75	−0.35	198.4 ± 1.82	0.58	0.11
	Spinal Cord	LQC (6.25 ng/g)	6.27 ± 0.17	2.70	0.46	6.11 ± 0.13	2.18	−2.18
		MQC (25 ng/g)	24.82 ± 0.51	2.06	−0.68	24.32 ± 0.65	2.68	−2.71
		HQC (200 ng/g)	200.2 ± 1.15	0.58	0.11	197.6 ± 0.76	0.38	−1.17
PMX205	Plasma	LQC (6.25 ng/ml)	6.2 ± 0.10	1.72	−0.80	6.3 ± 0.11	1.72	1.46
		MQC (25 ng/ml)	24.7 ± 1.06	4.31	−0.93	25.2 ± 1.09	4.31	0.96
		HQC (200 ng/ml)	198.7 ± 3.01	1.52	−0.64	202.6 ± 3.07	1.52	1.29
	Brain	LQC (6.25 ng/g)	6.37 ± 0.08	1.29	1.98	6.5 ± 0.25	3.85	4.12
		MQC (25 ng/g)	25.1 ± 1.15	4.57	0.37	24.7 ± 0.94	3.78	−1.07
		HQC (200 ng/g)	203.2 ± 3.04	1.50	1.58	198.1 ± 1.84	0.93	−0.94
	Spinal Cord	LQC (6.25 ng/g)	6.23 ± 0.08	1.29	−0.29	6.40 ± 0.06	1.00	2.41
		MQC (25 ng/g)	24.6 ± 1.12	4.57	−1.51	25.3 ± 1.1	4.33	1.51
		HQC (200 ng/g)	199.3 ± 2.3	1.50	−0.35	202.9 ± 3.5	1.71	1.50

Intra-day and inter-day precision and accuracy of the LC-MS/MS method for PMX53 and PMX205 determination in mice plasma, brain and spinal cord (*n* = 3 days, 6 imitates per day).

RSD: relative standard deviation; RE: relative error.

### Process recovery, efficiency and matrix effects

The process efficiency, extraction efficiency and recovery values as summarized in Table 3 suggested that the sample preparation results in exceptional extraction of analytes from various biological matrices with minimum matrix effect. It should be noted however, that the matrix effect was compensated for by the use of the structurally similar analogue (i.e. PMX53 or PMX205) as the internal quantitative standards during sample preparation and analysis.

**Table 3:** Process suitability and matrix influence for PMX53 and PMX205 determination.

	Spiked conc. plasma (ng/ml) & tissue (ng/g)	Process efficiency	Plasma			Brain			Spinal cord		
			Recovery	Extraction efficiency	Matrix effect	Recovery	Extraction efficiency	Matrix effect	Recovery	Extraction efficiency	Matrix effect
		(mean ± SD%)	(mean ± SD%)	(mean ± SD%)	(mean ± SD%)	(mean ± SD%)	(mean ± SD%)	(mean ± SD%)	(mean ± SD%)	(mean ± SD%)	(mean ± SD%)
PMX53	LQC (6.25)	98.1 ± 2.4	95.9 ± 0.4	95.3 ± 2.3	0.6 ± 0.5	97.2 ± 1.4	96.3 ± 3.3	−2.8 ± 4.8	96.3 ± 1.4	97.1 ± 1.4	2.2 ± 0.1
	MQC (25)	94.2 ± 2.4	94.7 ± 2.3	97.2 ± 2.3	2.6 ± 0.5	99.1 ± 3.4	98.2 ± 3.9	−0.85 ± 4.9	95.1 ± 1.4	99.1 ± 1.5	4.24 ± 1
	HQC (200)	98.2 ± 0.6	99.1 ± 1.9	98.1 ± 1.9	−0.94 ± 0.8	85.2 ± 0.4	84.6 ± 0.4	−0.67 ± 0.7	99.1 ± 1.5	98.3 ± 1.5	−0.7 ± 1.1
PMX205	LQC (6.25)	94.1 ± 0.7	92.5 ± 0.7	95.1 ± 0.8	2.4 ± 0.2	85.9 ± 1.1	87.4 ± 0.9	−2.0 ± 3.2	93.2 ± 6.2	95.4 ± 1.7	3.6 ± 0.5
	MQC (25)	93.4 ± 1.1	93.9 ± 2.2	96.5 ± 2.2	2.6 ± 0.5	87.1 ± 1.7	86.2 ± 1.6	−0.8 ± 4.5	94.1 ± 5.5	98.1 ± 5.8	4.2 ± 0.9
	HQC (200)	96.1 ± 0.9	90.6 ± 1.2	87.5 ± 1.2	−3.5 ± 3.7	97.1 ± 5.1	99.5 ± 5.2	3.5 ± 1.2	93.8 ± 4.1	90.4 ± 3.9	3.6 ± 3.7

Recoveries, extraction efficiencies and matrix effect of the bioanalytical method for PMX53 and PMX205 determination in mice plasma, brain & spinal cord (*n* = 3, 6 replicates per day).

### Stability studies

Stability studies were performed in the biological matrices, plasma, brain and spinal cord, to reflect and identify any degradation of PMX53 and PMX205 during the entire period of sample collection, storage, processing and analysis. The main aim of this method development was to apply a validated protocol for pharmacokinetic studies in mice not only by the *i*.*v*. route, but through various other routes of drug administration that involve an array of biological fluids such as the serum, gastric and intestinal environments. For these cyclic peptidic drugs (PMX53 and PMX205) it was important to identify *in vivo* metabolic stability responsible for duration of action in circulation, absorption from gut, and gastric stability, which may reflect oral activity. Hence, in addition to storage and post-preparative stability, metabolic stability of both analytes was analysed in serum, gastric and intestinal environments.

#### Storage and post-preparative stability

Results, as expressed in Table 4, represent the storage stability of analytes in biological matrices. The stability of PMX53 and PMX205 in plasma, brain and spinal cord matrices stored for four hours at room temperature, in −20 ± 5 °C storage conditions for up to twelve months and after three freeze-thaw cycles were within an acceptable range of guidelines (i.e. ±15% for medium QC (MQC) and high QC (HQC) samples. For LQC samples, ±25% criteria with a minimum of three values within the range of ±20% was used as per regulatory guidelines). Further stability could potentially be improved by reducing storage conditions from −20 ± 5 °C to −80 ± 5 °C. Supplementary Table 2, represents the high stock solution stability of both analytes up to six months in the current storage conditions. Results of post-preparative stability of PMX53 and PMX205 as determined by performing auto-sampler stability, auto-sampler reproducibility and comparing the results of processed samples with unprocessed standard samples, support the reliability of developed method. In summary, the combined results reflect the stability of PMX53 and PMX205 under post-preparative conditions and the reliability of conditions for analyte quantification.

**Table 4:** Stability studies for PMX53 and PMX205.

	Storage conditions	Spiked conc. plasma (ng/ml) & tissue (ng/g)	Plasma (*n* = 6)			Brain (*n* = 6)			Spinal cord (*n* = 6)		
			Measured conc. (ng/ml) (mean ± SD)	Precision (RSD, %)	Accuracy (RE, %)	Measured conc. (ng/g) (mean ± SD)	Precision (RSD, %)	Accuracy (RE, %)	Measured conc. (ng/g) (mean ± SD)	Precision (RSD, %)	Accuracy (RE, %)
PMX53	Short term (4 h at room temperature)	LQC (6.25)	6.24 ± 0.34	5.48	−0.20	6.28 ± 0.97	15.46	−0.06	6.56 ± 0.32	4.94	4.72
		MQC (25)	24.81 ± 0.56	2.28	−0.78	24.9 ± 0.25	0.99	−0.46	24.9 ± 0.62	2.47	−0.49
		HQC (200)	199.13 ± 2.71	1.36	−0.44	199.3 ± 3.6	1.81	−0.34	199.2 ± 3.1	1.57	−0.41
	Freeze – thaw stability (3 cycles)	LQC (6.25)	6.24 ± 0.56	8.98	−0.18	6.36 ± 0.18	2.76	1.75	6.49 ± 0.86	13.20	3.64
		MQC (25)	24.9 ± 0.31	1.25	−0.41	24.8 ± 0.63	2.54	−0.74	24.9 ± 0.16	0.63	−0.12
		HQC (200)	199.97 ± 4.38	2.19	−0.01	199.8 ± 3.1	1.52	−0.42	199.7 ± 3.9	1.96	−0.16
	Long term (−20 °C for 12 months)	LQC (6.25)	6.22 ± 0.88	14.10	−0.46	6.40 ± 0.45	7.01	2.32	6.33 ± 0.19	2.94	1.27
		MQC (25)	24.88 ± 0.55	2.21	−0.50	24.9 ± 0.33	1.32	−0.24	24.9 ± 0.64	2.58	−0.20
		HQC (200)	199 ± 2.83	1.42	−0.50	200.7 ± 4.5	2.25	0.34	199.9 ± 3.5	1.50	−0.07
PMX205	Short term (4 h at room temperature)	LQC (6.25)	5.91 ± 0.24	4.05	−5.80	6.18 ± 1.22	19.71	−1.18	5.99 ± 0.27	4.58	−4.34
		MQC (25)	24.97 ± 1.28	5.14	−0.12	24.75 ± 1.71	6.84	0.56	25.07 ± 2.01	8.02	0.29
		HQC (200)	197.6 ± 2.16	1.10	−1.19	198.9 ± 7.74	3.87	0.84	200.1 ± 2.57	1.28	0.45
	Freeze – thaw stability (3 cycles)	LQC (6.25)	6.18 ± 0.81	13.16	−1.18	5.84 ± 0.20	3.34	−7.01	6.17 ± 1.36	22.07	−1.36
		MQC (25)	24.76 ± 1.55	6.26	−0.95	24.73 ± 1.27	5.15	−1.10	24.87 ± 1.88	7.56	−0.51
		HQC (200)	198.9 ± 2.9	1.46	−0.55	197.3 ± 2.24	1.13	−1.36	201.9 ± 6.86	3.40	0.95
	Long term (−20 °C for 12 months)	LQC (6.25)	6.05 ± 0.68	10.90	−0.01	5.86 ± 0.30	5.09	−6.59	5.89 ± 0.19	3.25	−6.17
		MQC (25)	24.8 ± 1.81	7.23	−0.01	24.32 ± 1.24	5.09	−2.79	24.82 ± 1.45	5.84	−0.72
		HQC (200)	199.9 ± 3.24	1.62	−0.01	199.7 ± 2.30	1.15	−0.13	197.4 ± 2.57	1.30	−1.31

Determination of PMX53 and PMX205 stability in different matrixes under different storage conditions.

RSD: relative standard deviation; RE: relative error.

#### Plasma and serum stability

**Figure 2:**
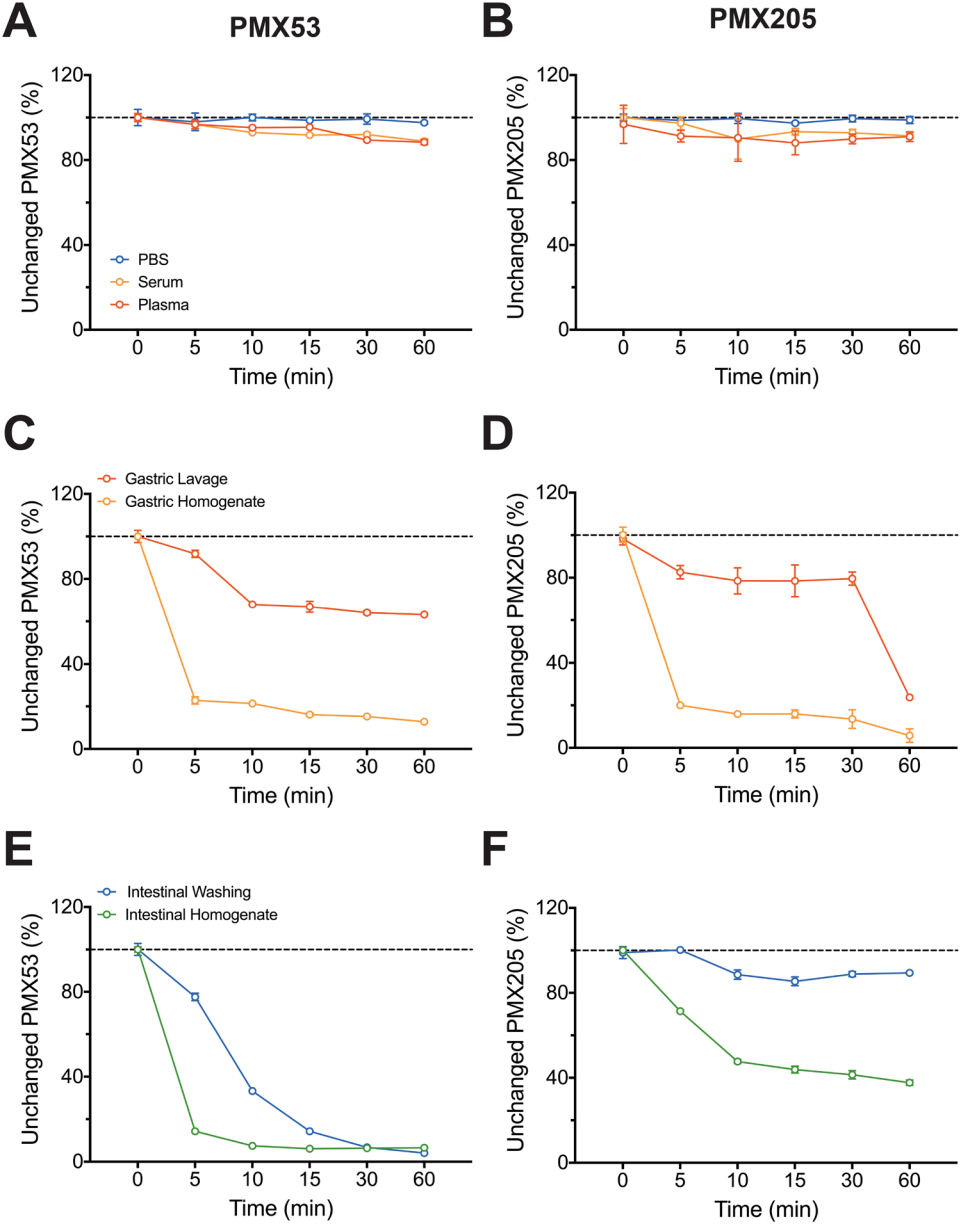
PMX53 and PMX205 stability studies: Stability study of PMX53 and PMX205 in serum, plasma, gastric and intestinal environment at 37 °C for 60 min (**A**–**F**). Line graph represents unchanged PMX53 and PMX205 at regular time intervals as determined by validated LC-MS/MS method. Data expressed as mean ± SEM of *n* = 4 of percentage of detected concentration compared to initial concentration values.

The results illustrate the stability of PMX53 and PMX205 in plasma (88 ± 1.2% and 91.4 ± 3.1%) and serum (88 ± 1.5% and 91.4 ± 1.4%) respectively when incubated at 37 °C for up to 60 min, and were comparable with stability of analytes in control solution (i.e. PBS) when stored and analysed using similar conditions (Fig. 2A,B**)**. In conclusion, during pharmacokinetic studies for up to 60 min, the amount of analytes detected reflects the unchanged form of PMX53 and PMX205 in plasma and serum.

#### Gastric environment stability

Figure 2C,D illustrates the gastric stability of PMX53 and PMX205. The stability of the antagonists was expressed as a percentage of concentration detected at different time points in comparison to concentration detected immediately following addition of antagonists, i.e. at t = 0 min. For PMX53 and PMX205, the proteinaceous nature of analytes resulted in some degradation in the gastric environment. PMX53 was more stable in gastric lavage fluids with more than 60% of unchanged form compared to PMX205 (<25%) for up to 60 min. Overall, the results illustrate the low stability of PMX53 and PMX205 in mouse gastric lavage (63.2 ± 0.27% and 23.3 ± 1.7%) and gastric homogenates (12.9 ± 0.23% and 6.7 ± 4.2%) respectively when incubated at 37 °C for up to 60 min.

#### Intestinal environment stability

Figure 2E,F illustrates the intestinal stability of PMX53 and PMX205 when incubated at 37 °C for up to 60 min. The intestinal stability of PMX205 was far better in comparison to PMX53 with more than 85% of unchanged form in intestinal washing and greater than 35% in intestinal homogenates for up to 60 min. By comparison, PMX53 has very low stability in intestinal washing and intestinal homogenates (<7%) respectively when incubated at 37 °C for up to 60 min. This mirrors prior stability studies in rat intestinal homogenates indicating that PMX205 has greater intestinal stability over PMX53^15^.

### Carryover effect and dilution integrity

During the validation and analysis studies, no significant carry over effect was observed for both PMX53 and PMX205. Absence of chromatographic peaks of PMX53 and PMX205 in mobile phase (zero analyte) chromatograms following injections containing the highest concentration of analyte, reflect the reliability of auto sampler, column and method for LC-MS/MS analysis. The accuracy and deviation results of dilution for PMX53 in various matrices was greater than 98.1% and less than 2.4%, and for PMX205 was greater than 97.5% and less than 3.2%, indicating the reliability of the dilution method for quantification of analytes in samples containing analytes outside the range of calibration standard curve.

### Pharmacokinetic studies

**Figure 3:**
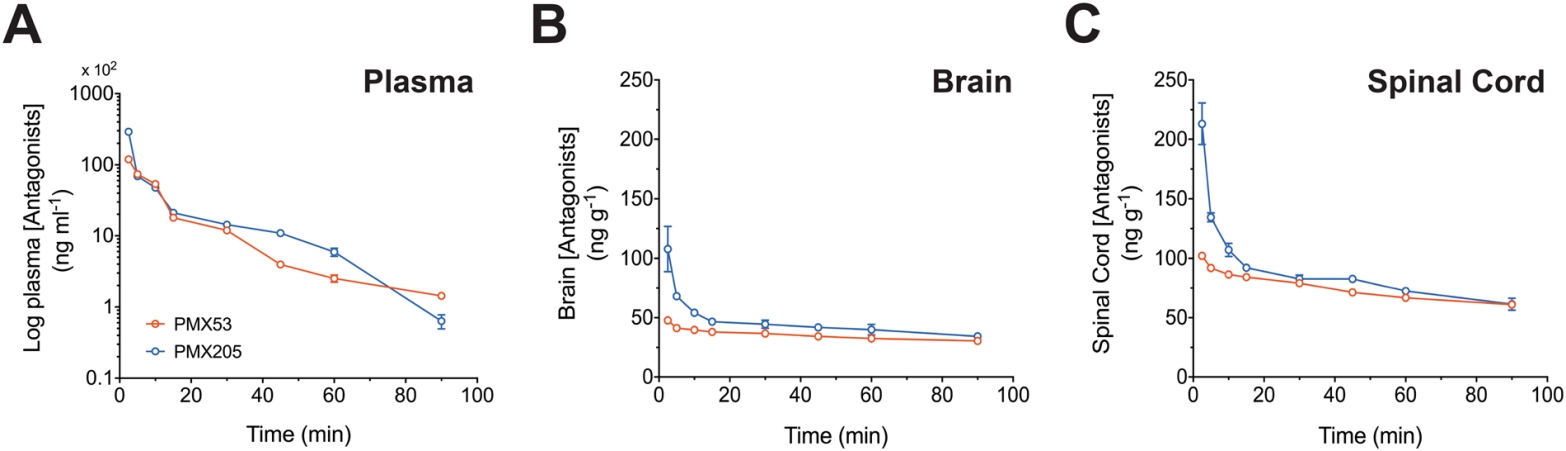
Intravenous (*i*.*v*.) pharmacokinetics of PMX53 and PMX205 in wild-type mice: Complement C5a receptor 1 antagonists PMX53 (red graph lines) and PMX205 (blue graph lines) concentration vs time profile in plasma (**A**), brain (**B**) and spinal cord (**C**) following single *i*.*v*. bolus drug dose of 1 mg/kg of either antagonist in mice at time = 0. Data points represent mean ± SEM of *n* = 4 mice at each time point.

The suitability and reliability of the bio-analytical LC-MS/MS method was finally assessed by its application to pharmacokinetic studies in mice. The pharmacokinetics of PMX53 and PMX205 were performed by quantitative determination of plasma, brain and spinal cord levels of PMX53 and PMX205 at different time points following a single *i*.*v*. administration of drug. The mean plasma, brain and spinal cord concentrations versus time profiles of PMX53 and PMX205 following an *i*.*v*. bolus dose of 1 mg/kg is shown in Fig. 3, and the corresponding pharmacokinetics parameters as determined by non-compartmental analysis using Pharsight WINNONLIN software are summarized in Table 5. The pharmacokinetic parameters and concentration-time profiles indicate a fast distribution and elimination of PMX53 and PMX205, as reflected by significant reductions in the blood immediately after administration. Additionally, the half-life and mean residence time of PMX53 and PMX205 are relatively short (Table 5). PMX205 demonstrated overall higher concentrations in blood, brain and spinal cord, compared to PMX53, as reflected by its higher Cmax values. However, PMX205 also displayed faster clearance compared to PMX53, potentially due to its more lipophilic nature. The plasma and tissue volume of distribution of PMX53 was also higher compared to PMX205, however PMX205 had higher absolute concentrations in the brain and spinal cord over the time-frame examined. These results confirm the suitability and reliability of the developed LC-MS/MS method for the quantitative determination of PMX53 and PMX205 in plasma, brain and spinal cord tissue of mice.

**Table 5:** PMX53 and PMX205 pharmacokinetic parameters.

Parameter	PMX53			PMX205		
	Plasma	Brain	Spinal cord	Plasma	Brain	Spinal cord
Cmax (μg/ml or ng/g)	11.9	47.7	102.07	29.03	107.81	213.1
t1/2 (hr)	0.52	3.79	3.40	0.17	2.69	1.78
AUC 0-t (min*μg/ml or g)	204.6	3.18	6.69	347.90	4.18	7.95
AUC 0-inf_obs (min*μg/ml or g)	229.6	13.21	24.67	348.86	12.18	17.42
MRT 0-inf_obs (hr)	0.30	5.46	4.86	0.15	3.75	2.49
Vz_obs (ml or g)	8.26	745.1	358.60	1.32	572.84	265.65
Cl_obs (ml/min or g/min)	0.18	2.27	1.21	0.086	2.46	1.722
Vss_obs (ml or g)	3.28	743.9	354.73	0.79	553.84	257.98

Pharmacokinetic parameters of PMX53 and PMX205 after single intravenous administration of 1 mg/kg of complement C5a receptor 1 antagonist to wild-type mice.

Values represent calculated parameter values using mean of *n* = 4 concentration values. C max, max concentration of antagonist; t ½, elimination half-life; AUC 0-t, area under the concentration-time curve from time zero to time t i.e. 90 min; AUC 0-infinity, area under the concentration-time curve from time zero to infinity; MRT, mean residence time; Vz, volume of distribution at terminal phase; Cl, clearance; Vss, volume of distribution at steady state.

## Discussion

PMX53 and PMX205 are two widely used antagonists of complement C5aR1. They are commonly used in mouse models of inflammatory disease and have recently shown promise as potential therapeutics for neurodegenerative diseases^4,7,18^. Despite their widespread use in mice, there is limited information on their pharmacokinetic profile in this species. Performing pharmacokinetics of a drug following various routes of administration is a major requirement for preclinical drug development progression, and is essential for the determination of drug bioavailability and key pharmacological parameters. In order to perform proper and standardized pharmacokinetic of a drug, the main requirement is the development of a sensitive, reproducible and validated quantitative bio-analytical method. LC-MS/MS techniques are widely utilized for quantitative purposes especially in pharmacokinetic studies due to their high levels of sensitivity. Hence, a robust, accurate, sensitive and specific bioanalytical LC-MS/MS method for quantitative determination of PMX53 and PMX205 in mice plasma, brain and spinal cord was established and validated according to US FDA and European Medicines Agency guidelines^23,24^. In comparison to previously published LC-MS/MS methods^7,13,15,21,22^, the present method demonstrated superior extraction and process efficiency, using a simple and rapid protein precipitation method, together with a short chromatographic run time. The present method also had lower limits of detection (0.48 ng/ml for plasma and 1.56 ng/g in tissues) compared to previously published methods (3 ng/ml for plasma and 5 ng/g in tissues)^7,21^. The low LOD and LOQ values reflect the sensitivity of method at small volumes of extraction. Importantly, the results of the reported validation parameters and method applicability support the reliability of this LC-MS/MS method for the analysis of large sample batches without any instrument performance loss.

For drugs, especially peptidic drugs such as PMX53 and PMX205, the *in vivo* stability in serum, plasma, gastric and intestinal conditions strongly influence the resulting pharmacokinetic profile. Gastric and intestinal transit time also influences the rate and extent of drug absorption following oral administration. Hence, stability studies of analytes are helpful in identifying and selecting a route of drug administration with desirable levels of circulating drug to achieve a maximum therapeutic effect. In the present study, the relatively low gastric and intestinal stability of both PMX53 and PMX205 indicates that oral drug administration may not be the most suitable route to achieve desirable therapeutic concentrations for future clinical translation. Indeed, prior pharmacokinetic studies in rats indicated that PMX53 had an oral bioavailability of ~5%^15^. Regardless, both drugs have demonstrated therapeutic benefits in various mice models following drinking water administration^4,18,25,26^, which could potentially reflect absorption through buccal, sublabial or sublingual sites, instead of intestinal/gastric absorption^21^. Alternatively, the relatively prolonged *in vivo* activity of the drug class due to their non-competitive antagonist pharmacology may also help explain their consistent oral activity *in vivo*^13^. Future studies comparing these various oral sites of administration for PMX53 and PMX205 in mice could help delineate this further.

In summary, this study developed a rapid, robust, selective, and highly sensitive method for the quantification of peptidic C5aR1 antagonists, supported by reproducibility, adequate recovery and minimal matrix effects. It was successfully applied to determine the preclinical pharmacokinetics and central nervous system tissue distribution of PMX53 and PMX205 in mice via the *i*.*v*. route of drug administration. This validated method can therefore be applied to further investigate the preclinical pharmacokinetics of these compounds in healthy mice, and in various models of mouse disease.

## Methods

### Chemicals and reagents

Complement C5aR1 antagonists, PMX53 (Ac-Phe-[Orn-Pro-dCha-Trp-Arg]); (Fig. 1A) and PMX205 (hydrocinnamate-[Orn-Pro-dCha-Trp-Arg]); (Fig. 1B) were kindly provided by Promics Pty Ltd, Australia, and were synthesized and characterised as previously described^5^. Acetonitrile (LCMS grade), methanol (AR grade) and ethanol (AR grade) were sourced from Ajax FineChem (Australia). Formic acid (optima LCMS grade) was purchased from Fisher Scientific (USA). Ultra-pure deionized water was obtained from Millipore Milli-Q water system (Millipore MA, USA). Mouse plasma containing EDTA as an anticoagulant was prepared in-house. Tissue samples (brain and spinal cord) were collected from animals following transcardial perfusion with sterile saline solution^27^.

### Instrumentation

The LC-MS/MS system consisted of an API 3200 (AB SCIEX) triple quadrupole QTRAP LC-MS/MS mass spectrometer containing Turbo V electrospray ionization (ESI) source system united with Genius AB-3G nitrogen gas generator (Peak Scientific). The mass spectrometer was coupled with Agilent 1200 series HPLC system (Agilent Technologies) equipped with a degasser, a column oven, a binary pump and temperature controlled auto sampler. The auto sampler was set at 4 °C and column oven temperature was maintained in the range of 25 ± 1 °C. The system control and data acquisition were executed by Analyst software (AB SCIEX, Applied Biosystems Inc., USA, version 1.5.1). Chromatographic separation was implemented by Kinetex EVO C18 analytical column (100 × 2.1 mm, 100 Å, 5 µm, Phenomenex Inc., CA, USA) under binary gradient conditions using mobile phase A (milliQ water containing 0.1% formic acid) and mobile phase B (acetonitrile containing 0.1% formic acid) with variable flow rate. The introduction of matrix components in the mass spectrometer was reduced by use of a diverter valve after 13 min of run. The injection volume of 10 µl was set for method development, validation and sample analysis. The column and auto sampler tray were maintained at 25 °C and 4 °C, respectively. Experimental data processing and analysis was performed by Multiquant software (AB SCIEX, USA, version 2.0), Microsoft Excel (Microsoft Inc., USA version 2013) and GraphPad Prism (LaJolla, CA, software version 7.0).

### Standard and quality control sample preparation

Stock solutions of PMX53 and PMX205 were prepared in 50% methanol at a target concentration of 1 mg/ml and serially diluted with 50% methanol to obtain stock working solutions at a concentration of 1000 ng/ml. All solutions were kept at −20 °C and brought to room temperature before use. Calibration standard samples were prepared by spiking working solution to mice plasma, brain and spinal cord homogenates from mice untreated with C5aR1 antagonists. Plasma, tissue calibration standard samples and quality control (QC) samples were processed with 10 µl of 1000 ng/ml internal standard (i.e. PMX205 as internal standard for PMX53 samples and PMX53 as internal standard for PMX205 samples). Samples were vortexed for 1 min and deproteinized with 1:3 ice cold LCMS grade acetonitrile. Samples were sonicated followed by centrifugation at 13,000 × *g* for 30 min for supernatant collection and then dried using centrivap sample concentrator (Labconco, VWR) at room temperature. Samples were stored at −80 °C for LCMS analysis. On the day of analysis, samples were resuspended in 50 µl of 75% methanol/water and 10 µl was analysed using the LC-MS/MS system.

### Method validation

The validation of bioanalytical method was performed for parameters such as selectivity, sensitivity, accuracy, precision, linearity, limit of detection, limit of quantification, stability and recovery in accordance with the guidelines of the US FDA^23^. Additionally, matrix effects, carry over and dilution integrity were also assessed to adhere with the guidelines for validation of bioanalytical methods by European Medicines Agency^24^.

### Selectivity and specificity

The LC-MS/MS method selectivity and specificity was evaluated to investigate the effect of endogenous interference in extracted samples. Processed blank samples from mice without any drug administration, matrix samples spiked with analytes at low quality control (LQC) concentrations, and samples obtained at 2.5 min from mice *i*.*v*. administered either PMX53 or PMX205, were used for selectivity and specificity studies.

### Calibration curve and sensitivity

Linearity of the calibration standard curve was assessed by plotting a peak area ratio (analyte/internal standard) of individual analytes in different matrices, versus applied concentrations on two consecutive days in replicates. Calibration standard samples of PMX53 and PMX205 were prepared by diluting working standard solutions in the matched-matrix along with internal standards to obtain ten calibration standards with a concentration range of 0.40–200 ng/ml for plasma, and eight calibration standards with a concentration range of 1.56–200 ng/g for brain and spinal cord homogenates. S/N script of Analyst software was used for identification of LOD values when signal noise was greater than 3.3 times the background noise of the matrix. LOD values were used as the lowest concentration of standard curves. GraphPad 7 software was utilized to calculate the slope, the intercept and the correlation coefficients by using linear regression and weighing factor of 1/x^2^. From the calibration curves, the sensitivity of the analytical method, i.e. the LLOD and LLOQ, was calculated for quantitative determination of PMX53 and PMX205 in different matrices. The LLOD and LLOQ were identified by using STEYX and slope values obtained from the standard curve using equations LLOD = (3.3 × STEYX)/slope and LLOQ = (10 × STEYX)/slope.

### Accuracy and precision

The intra-day and inter-day accuracy and precision of PMX53 and PMX205 were performed by analysing six replicates of QC samples on the same day and on three consecutive days. QC samples of both analytes were prepared and analysed individually at low (LQC), medium (MQC) and high (HQC) concentrations (6.25, 25 and 200) in different matrices using working stock solution dilutions and the results are expressed as relative standard deviation for accuracy and relative error for precision. FDA bioanalytical method validation guidelines were used as acceptability criteria for inclusion i.e. ±15% for accuracy and precision for QCs.

### Process recovery, efficiency and matrix effects

The overall deproteination and extraction process efficiency by 1:3 ice-cold acetonitrile was assessed through various parameters including process efficiency, extraction yield, recovery and matrix effect at LQC, MQC and HQC in various biological matrices. Process efficiency was evaluated by comparing the peak area of mobile phase spiked with QC concentration and processed as biological matrix samples to the peak area of respective QC standard solution. Extraction efficiency and recoveries were determined by comparing peak areas of spiked biological matrix to peak areas of standard solution and peak areas of processed biological matrix samples spiked with QC respectively. The % matrix effect was evaluated by comparing peak areas of processed QC sample with standard QC solution. The following equations were utilised for calculation of extraction efficiency, extraction recovery and matrix effect:

1 $${\rm{Extraction}}\,{\rm{efficiency}}=({\rm{BE}}/{\rm{C}})\,\ast \,100$$

2 $${\rm{Extraction}}\,{\rm{recovery}}=({\rm{BE}}/{\rm{AE}})\,\ast \,100$$

3 $${\rm{Matrix}}\,{\rm{effect}}=(1-({\rm{AE}}/{\rm{C}}))\,\ast \,100$$

where BE represents biological matrix samples spiked with QC before extraction, C represents matrix free solvent samples spiked with QC and AE represents biological matrix samples spiked with QC after extraction procedure.

### Stability studies

Stability studies were performed in six replicates at LQC, MQC and HQC levels in different matrices under various storage conditions. Short and long-term stability was evaluated by storing QCs in biological matrix for at least four hours at room temperature or −20 ± 5 °C storage conditions for up to twelve months. Freeze-thaw stability of QC samples in biological matrices was assessed at first and third freeze-thaw cycles stored at −20 °C. Six replicates of QC samples were processed and analysed along with freshly prepared QC samples in biological matrix to represent the stability of both analytes for up to three freeze-thaw cycles in plasma, brain and spinal cord matrices. Stock solution stability was determined by comparing 6 replicates of non-extracted (biological matrix-free) freshly prepared standard samples, with the standard samples stored at −20 ± 5 °C for up to six months. Post-preparative stability of PMX53 and PMX205 was determined by performing auto-sampler stability, auto-sampler reproducibility and comparing the results of processed samples with unprocessed standard samples. For auto-sampler stability and reproducibility, results from freshly prepared QC samples and QC samples stored for 48 hours at 4 °C were compared to represent auto sampler storage conditions. Process stability of PMX53 and PMX205 was performed to study the influence of the drying process. On the day of analysis, samples were processed and analysed along with freshly prepared QC samples in same matrix. According to the guidelines, ±15% is selected as acceptable stability criteria for samples/analytes to be considered as stable in MQC and HQC samples. However, for LQC samples ± 25% criteria is used with consideration that minimum of three values should be in the range of ±20% as required by regulatory guidelines.

Plasma and serum stability was determined by incubation experiments. For plasma stability, whole blood was collected via cardiac puncture from anaesthetized wild-type mice in tubes containing EDTA, and plasma was extracted by centrifuging blood for 15 min at 1500 × g at 4 °C. PMX53 and PMX205 plasma stability was performed by incubating 1 µg/ml of analyte stock solution in plasma at 37 °C for up to 60 min. Samples (*n* = 4) were quenched at regular intervals of 5 min, 10 min, 15 min, 30 min and 60 min by stopping the reaction with the addition of ice cold acetonitrile, and processed as described earlier for quantitative determination of unchanged analytes. For serum stability, whole blood was collected via cardiac puncture from anaesthetized wild-type mice in tubes and kept on ice until completely clotted. Serum was collected by centrifuging clotted blood for 15 min at 1500 × g at 4 °C. The stability of PMX53 and PMX205 in serum was performed by a similar manner via incubating 1 µg/ml of analyte stock solution in serum at 37 °C for up to 60 min, and samples (*n* = 4) were quenched by the addition of ice-cold acetonitrile, processed as described earlier for quantitative determination of unchanged analytes. Briefly, on the day of analysis, samples were deproteinized with 1:3 ice cold acetonitrile and spiked with 10 µl of 100 ng/ml internal standard. Samples were vortexed, centrifuged and supernatants were dried by vacuum concentration at room temperature. Dried samples were reconstituted in 50 µl of 75% methanol and analysed using LC-MS/MS. The stability the C5aR1 antagonists were expressed as a percentage of the concentration detected at different time points in comparison to the concentration detected immediately following addition of antagonists i.e. at t = 0 min.

The gastric stability of analytes was performed using gastric contents of wild-type mice with free access to food and water overnight (i.e. modelling physiological conditions). Mice were anaesthetized and euthanized by cervical dislocation. The stomach was clamped and removed from the abdominal cavity, and the stomach cavity was repeatedly lavaged with sterile saline (5 ml in total) and the lavage fluid collected and combined. The stomach tissue was then homogenized with an equal volume of milliQ water. Lavage fluid and stomach homogenate were centrifuged (400 × *g*, 30 min at 4 °C) and the supernatant was collected and stored on ice. A solution of C5aR1 antagonist (1 µg/ml) were incubated at 37 °C for up to 60 minutes in gastric lavage fluid and gastric homogenate supernatant, and samples (*n* = 4) were quenched at regular time intervals by the addition of ice-cold acetonitrile. Samples were processed as described earlier for the quantitative determination of unchanged analytes along with an internal standard.

Likewise gastric environment stability, intestinal stability of analytes was performed using intestinal contents of wild-type mice with free access to food and water overnight. Mice were anaesthetized and euthanized by cervical dislocation. The small intestine was clamped and removed from the abdominal cavity. The intestine was repeatedly lavaged with sterile saline (5 ml in total) and intestinal washings were collected and combined. The intestine was also homogenized with an equal volume of milliQ water. Intestinal lavage and intestinal homogenates were centrifuged (400 × *g*, 30 min at 4 °C) and the supernatant was collected and stored on ice. A solution of C5aR1 antagonist (1 µg/ml) was incubated at 37 °C for up to 60 minutes in intestinal lavage and intestinal homogenate supernatants, and samples (n = 4) were collected at regular time intervals by the addition of ice-cold acetonitrile. Samples were processed as described earlier for quantitative determination of unchanged analytes along with internal standard.

### Carryover effect and dilution integrity

The potential carry-over effect from the auto sampler and column was evaluated during the validation process through successive injections of the highest concentration i.e. 1000 ng/ml of PMX53 and PMX205 respectively during the start of method validation and in-between the runs followed by injections of samples containing only solvents of mobile phase. Pharmacokinetic samples might potentially contain analyte concentrations out of the calibration curve range. Hence, the effect of dilution was investigated in triplicates using 1 in 10 dilution of the matrix samples containing highest concentration of analyte (i.e. 1000 ng/ml for plasma and 1000 ng/g in tissue samples) with blank matrices to obtain the samples containing analytes within range of calibration curve.

### Application to pharmacokinetic studies

The validity of the developed bioanalytical LC-MS/MS method was assessed by quantitative determination of C5aR1 antagonist levels in mice plasma and tissue following *i*.*v*. drug administration. All experimental procedures and animal care were performed following approval from the University of Queensland Animal Care and Use Committee and conducted in accordance with the National Health and Medical Research Council of Australia policies and guidelines for the care and use of animals for scientific purposes. C57BL/6 J mice (males, 10–12 weeks old) were purchased from Animal Resources Centre, Western Australia. An *i*.*v*. dose of 1 mg/kg was selected based upon previous pharmacokinetic studies in rat and *in vivo* efficacy studies in mice^6,28^. After overnight fasting, mice (*n* = 4 per time point) were anaesthetized with zolazapam (50 mg/kg) and xylazine (12 mg/kg) via *i*.*p*. injection. Anaesthetized mice were *i*.*v*. administered 1 mg/kg of PMX53 or PMX205 in water for injection containing 5% ethanol through the tail vein. Blood samples were collected via cardiac puncture at 2.5 min, 5 min, 10 min, 15 min, 30 min, 45 min, 60 min and 90 minutes in tubes containing EDTA followed by plasma separation by centrifugation at 1500 × *g* at 4 °C. Plasma samples were stored at −80 °C for further processing and analysis. Mice were immediately perfused transcardially with sterile saline to remove circulating drug from the blood vasculature. Whole brain and spinal cord samples were obtained and homogenized in an equal weight-volume of milliQ water. 100 µl of tissue homogenate and 50 µl of plasma were mixed with 10 µl internal standard and processed similar to calibration standard samples for quantitative analysis of PMX53 and PMX205. Pharmacokinetics data analysis was performed using Pharsight WINNONLIN software (version 6.4) by a non-compartmental analysis method to obtain various pharmacokinetic parameters.

### Ethical approval and informed consent

All experimental procedures were approved by the University of Queensland Animal Ethics Committee and complied with the policies and regulations regarding animal experimentation. They were conducted in accordance with the Queensland Government Animal Research Act 2001, associated Animal Care and Protection Regulations (2002 and 2008), and the Australian Code of Practice for the Care and Use of Animals for Scientific Purposes, 8th Edition (National Health and Medical Research Council, 2013).

## Electronic supplementary material

Supplementary Information
