# Supplementary material for: Development and validation of a LC-MS/MS assay for pharmacokinetic studies of complement C5a receptor antagonists PMX53 and PMX205 in mice
Source: Sci Rep. 2018 May 25;8:8101. doi: 10.1038/s41598-018-26387-4 (PMC5970165; doi:10.1038/s41598-018-26387-4)
Supplement: Supplementary file 1 — Supplementary Information [file 41598_2018_26387_MOESM1_ESM.docx]

**Supplementary information**

**Development and validation of a LC-MS/MS assay for pharmacokinetic studies of complement C5a receptor antagonists PMX53 and PMX205 in mice**

Vinod Kumar^1^, John D. Lee^1, 2^ Richard J. Clark^1^, and Trent M. Woodruff^1*^

^1^School of Biomedical Sciences, the University of Queensland, Brisbane, QLD, 4072, Australia

^2^University of Queensland Centre for Clinical Research, the University of Queensland, Brisbane, QLD, 4029, Australia

**Supplementary Table 1: Sensitivity and linearity of calibration curves**

PMX53 and PMX205 calibration curves, lower limit of detection (LLOD) and lower limit of quantification (LLOQ) in biological matrixes

(*n* = 2 days, 3 replicates per day)

| Analyte | Biological matrix | Slope | y-intercept | R^2^ | Sensitivity | |
| --- | --- | --- | --- | --- | --- | --- |
|  |  | **(mean ± SD)** | **(mean ± SD)** | **(min-max)** | **LLOD** | **LLOQ** |
| PMX53 | Plasma | 0.0071 ± 9.573e-005 | 0.2115 ± 0.0399 | 0.9945-0.9962 | 1.76 ng/ml | 5.35 ng/ml |
|  | Brain | 0.0263 ± 0.0004 | 0.2115 ± 0.0399 | 0.9948-0.9996 | 2.75 ng/g | 5.79 ng/g |
|  | Spinal Cord | 0.0033 ± 2.063e-005 | 0.0148 ± 0.0009 | 0.9967-0.9994 | 3.16 ng/g | 6.31 ng/g |
| PMX205 | Plasma | 0.0026 ± 2.365e-005 | 0.0019 ± 0.0003 | 0.9972-0.9981 | 1.23 ng/ml | 3.73 ng/ml |
|  | Brain | 0.0138 ± 0.0001 | 0.0162 ± 0.0071 | 0.9939-0.9982 | 2.44 ng/g | 3.28 ng/g |
|  | Spinal Cord | 0.0078 ± 0.0001 | 0.0193 ± 0.0034 | 0.9855-0.9986 | 1.95 ng/g | 5.9 ng/g |

**Supplementary Table 2: Stock solution stability of PMX53 and PMX205**

| Spiked conc. | PMX53 (-20°C; up to 6 months) (*n* = 6) | | | PMX205 (-20°C; up to 6 months) (*n* = 6) | | |
| --- | --- | --- | --- | --- | --- | --- |
|  | **Measured conc. (ng/ml) (mean ± SD)** | **Precision (RSD, %)** | **Accuracy (RE, %)** | **Measured conc. (ng/ml) (mean ± SD)** | **Precision (RSD, %)** | **Accuracy (RE, %)** |
| LQC (6.25 ng/ml) | 6.21 ± 0.47 | 7.56 | -0.71 | 6.22 ± 0.73 | 11.71 | -0.41 |
| MQC (25 ng/ml) | 24.8 ± 0.74 | 3.00 | -0.79 | 24.82 ± 0.86 | 3.48 | -0.72 |
| HQC (200 ng/ml) | 200 ± 5.2 | 2.60 | 0.00 | 199.9 ± 4.87 | 2.43 | -0.01 |

RSD: relative standard deviation; RE: relative error


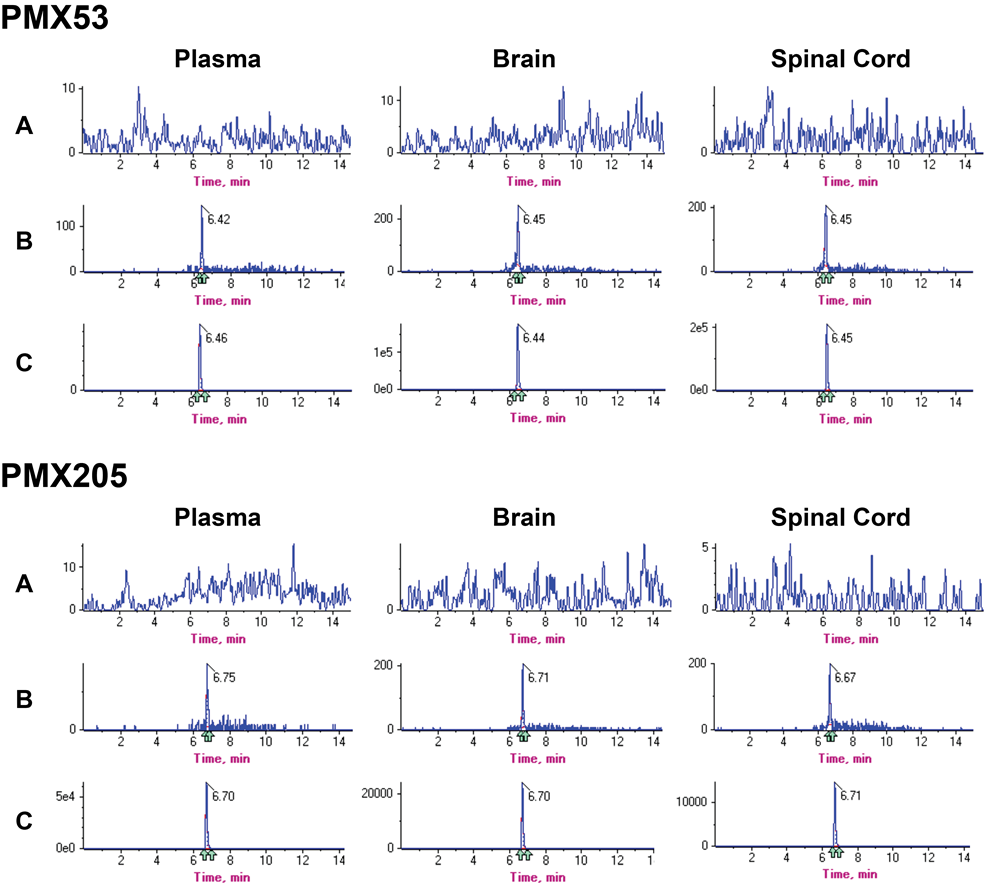


**Supplementary Figure 1:** Representative LC-MS/MS MRM chromatograms for PMX53 (m/z 448.6-120.2) and PMX205 (m/z 420.2-70.0) from (**A**) Double blank samples obtained and processed from mice untreated with PMX53. (**B**) Spiked sample chromatograms represent blank samples spiked with LQC (6.25) processed and analyzed. (**C**) Sample chromatograms represent samples obtained at 2.5 min from mice intravenously administered with 1 mg/kg of PMX53 through tail vein and processed for analysis.


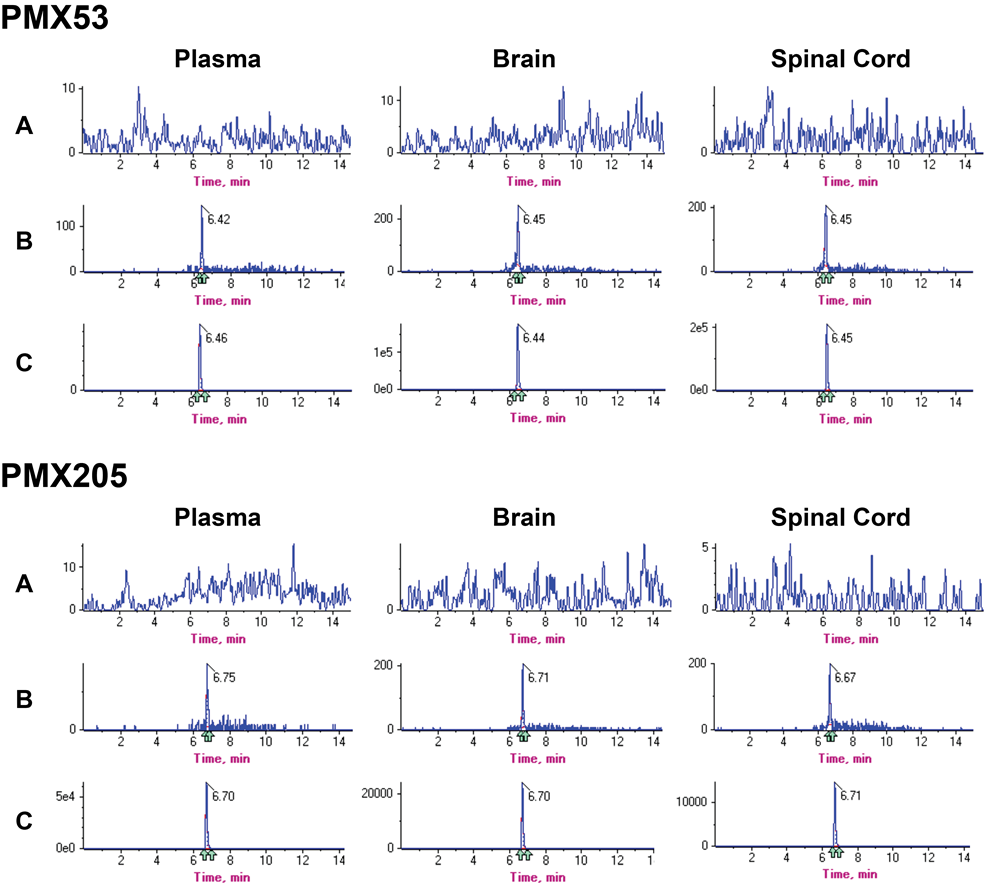


**Supplementary Figure 2:** Representative LC-MS/MS MRM chromatograms for PMX205 (m/z 420.2-70.0) from (**A**) Double blank samples obtained and processed from mice untreated with PMX205. (**B**) Spiked sample chromatograms represent blank samples spiked with LQC (6.25) processed and analyzed. (**C**) Sample chromatograms represent samples obtained at 2.5 min from mice intravenously administered with 1 mg/kg of PMX205 through tail vein and processed for analysis.
